# A Cyclic Nucleotide-Gated Channel Mutation Associated with Canine Daylight Blindness Provides Insight into a Role for the S2 Segment *Tri-Asp motif* in Channel Biogenesis

**DOI:** 10.1371/journal.pone.0088768

**Published:** 2014-02-21

**Authors:** Naoto Tanaka, Lucie Delemotte, Michael L. Klein, András M. Komáromy, Jacqueline C. Tanaka

**Affiliations:** 1 Department of Biology, Temple University, Philadelphia, Pennsylvania, United States of America; 2 Institute of Computational and Molecular Science, Temple University, Philadelphia, Pennsylvania, United States of America; 3 Department of Small Animal Clinical Sciences, College of Veterinary Medicine, Michigan State University, East Lansing, Michigan, United States of America; 4 Department of Clinical Studies, School of Veterinary Medicine, University of Pennsylvania, Philadelphia, Pennsylvania, United States of America; Monell Chemical Senses Center, United States of America

## Abstract

Cone cyclic nucleotide-gated channels are tetramers formed by CNGA3 and CNGB3 subunits; CNGA3 subunits function as homotetrameric channels but CNGB3 exhibits channel function only when co-expressed with CNGA3. An aspartatic acid (Asp) to asparagine (Asn) missense mutation at position 262 in the canine CNGB3 (D262N) subunit results in loss of cone function (daylight blindness), suggesting an important role for this aspartic acid residue in channel biogenesis and/or function. Asp 262 is located in a conserved region of the second transmembrane segment containing three Asp residues designated the *Tri-Asp motif*. This motif is conserved in all CNG channels. Here we examine mutations in canine CNGA3 homomeric channels using a combination of experimental and computational approaches. Mutations of these conserved Asp residues result in the absence of nucleotide-activated currents in heterologous expression. A fluorescent tag on *CNGA3* shows mislocalization of mutant channels. Co-expressing CNGB3 *Tri-Asp* mutants with wild type CNGA3 results in some functional channels, however, their electrophysiological characterization matches the properties of homomeric CNGA3 channels. This failure to record heteromeric currents suggests that Asp/Asn mutations affect heteromeric subunit assembly. A homology model of S1–S6 of the CNGA3 channel was generated and relaxed in a membrane using molecular dynamics simulations. The model predicts that the *Tri-Asp motif* is involved in non-specific salt bridge pairings with positive residues of S3/S4. We propose that the D262N mutation in dogs with *CNGB3*-day blindness results in the loss of these inter-helical interactions altering the electrostatic equilibrium within in the S1–S4 bundle. Because residues analogous to *Tri-Asp* in the voltage-gated Shaker potassium channel family were implicated in monomer folding, we hypothesize that destabilizing these electrostatic interactions impairs the monomer folding state in D262N mutant CNG channels during biogenesis.

## Introduction

Cyclic nucleotide-gated (CNG) channels in retinal rod and cone photoreceptor outer segments open in response to the binding of intracellular cGMP. Both Na^+^ and Ca^2+^ enter the outer segments through CNG channels; Na^+^ is the major current carrying ion while Ca^2+^ is a critical second messenger whose activity is closely monitored by many of the phototransduction enzymes as well as molecules involved in both light and dark adaptation [1]. CNG channels are non voltage-gated members of the tetrameric Shaker K^+^ channel superfamily. Each subunit has six transmembrane (TM)-spanning helices (S1 through S6), a pore region formed by the assembly of S5 and S6 and an S1–S4 bundle [2] which corresponds to the voltage-sensor domains of Shaker K^+^ channels. The intracellular N-terminal regions of CNG channels are involved in trafficking through an interaction with C-termini [3], [4]; these domains contain regulatory sites specific to the channel function [5]–[8]. The cytoplasmic C-terminal region contains a C-linker bridging the S6 helix to the cGMP binding domain [9]. A C-terminal leucine zipper domain of CNG A-type subunits is involved in recruitment of the B-type subunits for heteromeric channel assembly [10]. Cone photoreceptor CNG channels are assembled from both CNGA3 (A3) and CNGB3 (B3) subunits. The stoichiometry of cone channels is still debated [3], [10], [11], while the stoichiometry of rod channels is established as 3∶1 CNGA1/CNGB1 [12]–[14].

Mutations in the genes encoding A3 and B3 subunits are the most common causes for achromatopsia, an autosomal recessive channelopathy resulting in incomplete or complete loss of cone function [15]–[17]. The disease is characterized by poor visual acuity, lack of color discrimination, photophobia, and pendular nystagmus. This phenotype has been attributed to defects in CNG channel subunit folding and assembly, cellular localization and/or channel function [18]–[21]. Day blindness, an achromatopsia-like phenotype, was identified in German shorthair pointer dogs with an Asp to Asn missense mutation at position 262 of the CNGB3 subunit (B3-D262N) [22]. The loss of cone function in the affected animals suggests that Asp262 plays a critical role in cone CNG channels. Based on a sequence alignment with Shaker-K^+^ channels, we find that D262 is located in the second TM segment (S2) within a short 11 amino acid region. Two acidic residues precede D262 (hereafter denoted as D3) with D252 denoted as D1 and D256 denoted as D2. This acidic region is conserved within the CNG channel family and extends to members of the Shaker K^+^ superfamily. Recognizing the importance of this pattern, we designate this region the *Tri-Asp motif*. This work investigates the role of the *Tri-Asp motif*, and especially of D262 in the loss of cone function.

To investigate the molecular properties of the B3-D262N mutation, we exploited the conservation of the *Tri-Asp motif* and the propensity of A3 subunit to express functional homomeric channels. Here we report the cloning of the canine *A3* (*cA3*) gene and investigate the electrophysiological properties of the cone CNG channels expressed in a human embryonic kidney cultured cell line. Although the sequence of the canine *B3* (*cB3*) gene was previously reported [22], attempts to clone the gene into a eukaryotic expression vector have been unsuccessful. To characterize the function of heteromeric A3+ B3 channels, we co-expressed cA3 with the human B3 (hB3) gene. The electrophysiological properties of homomeric cA3 CNG channel closely match those reported previously with human A3 (hA3) channels [6], [20], [23]–[26]. We investigated the relative permeability of Ca^2+^ to Na^+^ and we find a ∼10 fold increase in Ca^2+^ permeability when the hB3 subunit is co-expressed with cA3. A possible pathophysiological consequence of this difference in Ca^2+^ permeability is discussed.

The cA3 subunit contains a *Tri-Asp motif* equivalent to the canine missense mutation; Asp 231 is the residue equivalent to Asp 262 (D3) in the cB3 subunit. A mutant cA3-D3/N was generated to study this substitution in a homo-tetrameric channel; no channel activity was observed. Cellular localization was examined using a fluorescent tag (YFP) on the cA3-D3/N mutant subunit. The mutant subunits mislocalized to cellular aggregates consistent with a folding or assembly defect. Mutations of D1 or D2 in the *Tri-Asp motif* also result in a loss of channel function and mislocalization. A homology model of canine A3 relaxed using molecular dynamics (MD) simulations points to a role for the *Tri-Asp motif*, most particularly for D3, in the stabilization of the S1–S4 bundle through electrostatic interactions. We propose that all mutations in the Asp residues of the *Tri-Asp* region result in improper folding of the TM regions of the subunit destabilizing the bundle assembly.

## Materials and Methods

### Ethics Statement

All investigations conformed to the ARVO statement for the Use of Animals in Ophthalmic and Vision Research and were approved by the University of Pennsylvania Institutional Animal Care and Use Committee (protocol number (803429; Institutional NIH/PHS Animal Welfare Assurance number A3079-01).

### Cloning of *CNGA3* from Canine Retina

Whole retinas were dissected from eyes enucleated immediately postmortem from dogs. The retinas were immediately frozen in liquid nitrogen and maintained under cryogenic conditions until analyzed. Total RNA was isolated from retinal tissue using TRIzol (Invitrogen) and a single chloroform extraction. First strand cDNA was synthesized in 20 µl reactions containing 0.4 µM of both forward and reverse primers, 1.5 mM MgCl_2_, 50 mM KCl, 10 mM Tris-HCl, (pH 8.3), 200 µM dNTP, and 0.5 U Taq polymerase using an oligo d(T) 16 primer and the GeneAmp RNA PCR kit following the manufacturers recommendations (Perkin Elmer). Two pairs of canine A3 specific primers were designed to amplify the coding region of the gene. The full-length gene was cloned into pCR-TOPO-II**®** (Invitrogen) and sequenced. The sequence for *cA3* was submitted and is under review at GenBank (BankIt1673058 cCNGA3 KF806731). The canine *B3* sequence was determined previously [22] (GI: 50978664). Multiple attempts to clone the canine *B3* gene into a eukaryotic expression vector were unsuccessful.

### Cloning of Canine *A3* and Human *CNGB3* (*B3*) into Mammalian Expression Vector and Generation of Mutant Subunits

The *cA3* in the pCR-TOPO-II vector was restricted with BamHI (New England BioLabs) and EcoRI (New England BioLabs) restriction endonucleases. PCR primers were designed to introduce HindIII recognition site at the 5′ end of the gene and AgeI recognition site at the 3′end of the gene which eliminated the stop site. The PCR primers are 5′- CGGAAGCTTGCAGAGATGGCCAAGATTAACACCCAAGTCTCC-3′ and 5′-GGCGACCGGTGACTGCTCTTTGATCTCTGTTTTTGCGGC-3′. The pEYFP-N1 vector (Clonetech) was prepared for ligation by digesting with HindIII and AgeI. The PCR gene product was ligated into the pEYFP and the product was used to transform Library Efficiency® DH5α Competent Cells (Invitrogen). DNA sequencing confirmed the full length gene product in the pEYFP-N1vector. The *hB3* gene, a gift from Bernd Wissinger, was cloned into pCDNA 3.1 (Invitrogen) for eukaryotic expression. Attempts to clone this gene into pEYFP-C1 or pEYFP-N1 to examine the cellular localization of the fluorescent tag resulted in the failure of heteromeric channel expression when co-expressed with untagged cA3. For this reason, all heteromeric channel studies were comprised of cA3 in pEYFP co-expressed with human hB3 in pcDNA3.1. Mutations were generated using primers designed with point mutations; mutagenesis was accomplished using QuikChange® (Stratagene, San Diego, CA). Full-length sequences were obtained for all mutant cDNAs.

### Heterologous Expression and Electrophysiology of CNG Channel Currents

The human embryonic kidney cell line tSA201 was used for expression studies as previously described [20]. Cells were transfected with 2–3 µg of *cA3-YFP* gene or co-transfected with 1–2 µg of canine c*A3-YFP* gene and 2–4 µg *hB3* gene employing Lipofectamine 2000 (Invitrogen, Carlsbad, CA). Inside-out patches were isolated from transfected cells using glass electrodes with resistances of 1.2–4.2 MΩ; bath and electrode solutions contained 120 mM NaCl, 2 mM EDTA, 2 mM EGTA, and 5 mM Hepes at pH 7.4 unless otherwise noted. The electrode was detached from the cell and placed in the inflow stream of the solution bank. Concentrations of cGMP in the range of 1–500 µM and cAMP in the range of 100–10000 µM were prepared in the bath solution. Nucleotide solutions flowed into the chamber through an inflow tube connected to a solution bank thereby contacting the intracellular face of the excised patch [27]. A series of 300-ms voltage pulses were applied in 10-mV steps between −80 mV and 80 mV. Currents were recorded at room temperature with an amplifier (AXOPATCH 10) and digitizer (DIGIDATA 1322A) and were analyzed on computer software (Clampfit 10.0, Table curve, Sigmaplot). The net currents were obtained by subtracting the bath current in the absence of nucleotide from each cAMP or cGMP activated current. Current data were averaged from multiple traces and fitted by the Hill equation,
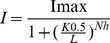



For L-cis-diltiazem and K_0.5_ values, net currents were normalized to the maximum cGMP activated current for each patch.

For Ca^2+^ reversal potential (E_Rev_) measurements, the bath solution was 120 mM NaCl and 5 mM Hepes at pH 7.4 with 1 mM Ca^2+^ added to the bath in the presence or absence of cGMP. A voltage-ramp was applied from −20 mV to +20 mV and back to −20 mV. The currents with ascending and descending ramps were digitally averaged and linearized to obtain a best fit to the zero current value or E_Rev_ [28]. The relative permeability ratio of calcium to sodium, P_Ca_/P_Na_, after single-sided addition of Ca^2+^, was determined according to the Goldman-Hodgkin-Katz current equation extended to include the E_Rev_ of permeant divalent cations and ignoring surface charge effects. Activity values for sodium and calcium were used as described previously [28] with the Lewis equation [29] as follows:
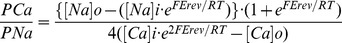



### Immunocytochemistry

Localization studies were performed as described previously [20]. 24 hours after transfection, cells on coverslips were washed with PBS 3 times and fixed with 4% paraformaldehyde in PBS for 20 minutes. After washing with PBS 3 times, the coverslips were mounted on slides using Vectashield® (Vector Laboratories, Inc.). Fluorescent cells were counted under 40x and photographed at 100x in a fluorescent microscope (Nikon). Unpaired t-tests were performed by QuickCalcs (http://www.graphpad.com).

### Amino Acid S1–S6 Sequence Alignments with Kv1.2/2.1

Amino acid sequences of cA3 (GI: 345777245), hA3 (GI: 4502917), cB3 (GI: 50978664), and hB3 subunit (GI: 116642889) were aligned with the Kv1.2/2.1 (GI: 16087792) sequence using Clustal X version 2.0.1. [30] (http://www.clustal.org/). The blocks of amino acid substitution matrix were used, together with standard parameters (opening gap penalty of 10, gap extension penalty of 0.20, and gap distance of 5). The S1–S6 TM domains were then assigned according to the crystal structure of Kv1.2/2.1 (2R9R).

### Homology Modeling

The procedure described here was previously used to build a model of the TM segments of Nav1.4 [31]. Briefly, the best template available to build a model of the TM domains of cA3 was identified by a PSI-BLAST search over the Protein Data Bank database [32]. Among the hits, the Kv1.2/2.1 high-resolution structure released in 2007 [32] as the candidate bearing the TM domains with the highest max score. A standard MODELLER [33] routine was then used to build a comparative model of the TM region of the cA3 channel, comprising the pore domain (S5–S6) and the four auxiliary domains (S1–S4, corresponding to the voltage-sensor domains in Kv1.2/2.1). Because the channel is a homotetramer, we enforced tetrameric symmetry between the Cα atoms of the four subunits. To direct the charged residues of the auxiliary domains away from the lipid/protein interface and have them point toward the hydrated lumen, we enforced distance restraints between pairs of positively/negatively charged residues in cA3: (K259(S3)/D221(S2), K294(S4)/D221(S3), K259(S3)/D225(S2), R297(S4)/D225(S2), R252(S3)/D231(S2), R235(S2)/D249(S3), K248(S3)/D303(S4), K255(S3)/E300(S4). These pairs were chosen after an initial unrestrained model was equilibrated with MD simulations (see below) and water molecules were seen to be protruding into the bilayer in response to the presence of bare charged residues. Thus, we identified the most probable contacting pairs while excluding those that would cause unfavorable conformations. To further avoid water protrusion within the membrane, a specific conformation of D398 (located at the extracellular end of S6) was chosen. We found that placing D398 in contact with polar S373 (pore loop) maintained the integrity of the membrane. The pair of contacting residues E306 (S4–S5 linker) and R424 (S6) was pulled together by enforcing distance restraints. The proline residue that defines the break in the S3 helix of Kv1.2/2.1 is absent in the CNG channels which likely indicates that S3 is entirely helical (no break between S3a and S3b as in the template). We therefore enforced helicity of the S3 segment.

### Molecular Dynamics (MD) Simulations

The homotetrameric cA3 channel was inserted in a fully hydrated palmitoyl-oleoyl-phosphatidyl-choline (POPC) bilayer (280 lipid molecules, 31,294 water molecules, 79 Cl^-^ ions, 71 Na^+^ ions, and the total size of the system is 147,992). The systems were equilibrated using the program NAMD2.9 [34]. The simulations were carried out under normal constant temperature and pressure conditions (298 K, 1 atm) in a 120 mM NaCl solution using the following scheme: The lipid tails were first melted while keeping the rest of the atoms of the system fixed during 300 ps. Then, to ensure correct reorganization of the lipids and solution, the positions of all of the atoms of the channel were constrained during 0.5 ns. In a third step, the side chains were allowed to reorganize while the backbone was kept restrained (5 ns with a 1 kcal/mol/Å^2^, 5 ns with a 0.5 kcal/mol/Å^2^ force constant and 5 ns with a 0.1 kcal/mol/Å^2^ force constant). At this stage, hydration of the membrane and the TM segments of the protein were prevented by applying a repulsive harmonic potential to the water molecules at the membrane/solution interface. For this channel, this step is crucial, due to the high number of charged residues in TM positions. Next, the channel was relaxed while keeping a harmonic constraint on the backbone of the S4 helix for 65 ns. Finally, a 65-ns unrestrained MD simulation of the entire channel was conducted, enabling full relaxation of the system. Langevin dynamics was applied to keep the temperature (300 K) fixed. The equations of motion were integrated using a multiple time-step algorithm [35]. Short- and long-range forces were calculated every 1 and 2 time-steps respectively, with a time step of 2.0 fs. Chemical bonds between hydrogen and heavy atoms were constrained to their equilibrium values. Long-range electrostatic forces were taken into account using the particle mesh Ewald approach [36]. The water molecules were described using the TIP3P model [37]. The simulation used the CHARMM22-CMAP force field with torsional cross-terms for the protein [38] and CHARMM27 for the phospholipids [39]. The simulations were performed on Owls’ Nest, Temple University’s supercomputing facilities.

## Results

### Canine CNG Channel Amino Acid Alignment and Predicted TM Topology

**Figure 1:**
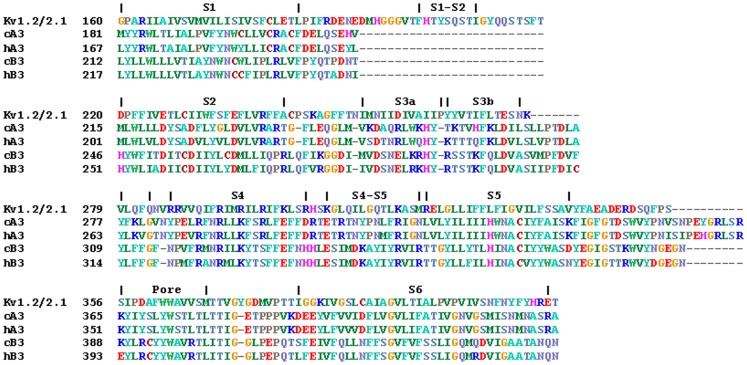
Predicted topology of the TM regions of canine and human A3 and B3 subunits. The TM domains are defined by the crystal structure of the chimeric voltage-gated potassium channel Kv1.2/2.1 (2R9R) [40]. The amino acid sequence identity between the cA3 and hA3 is 82% and is 76% between cB3 and hB3. c = canine, h = human.

To predict the topology of the CNG channel subunits, the amino acid sequence of the cA3 TM regions was used to search the entire protein sequence database. Using a PSI-Blast search, the chimeric voltage-gated K^+^ channel, Kv1.2/2.1 (2R9R) was identified as the closest match for a protein of known structure [40]. The S1–S6 TM regions of the cone CNG channel subunits then were aligned with the S1–S6 region of Kv1.2–2.1; the alignment is shown in Figure 1. The signature sequence of the K^+^ selectivity filter (TVGYG) is lost in CNG channels.

One striking difference between the cA3 and the template Kv1.2/2.1 is the larger number of charged residues located in the TM segments S1–S4 in the cA3 subunit. Specifically, S2–S4 bear 8 negatively charged residues (3 on S2, 2 on S3 and 3 on S4) and 12 positively charged residues (2 on S2, 5 on S3 and 5 on S4). It is worth noting that S1 only contains two positively charged residues, one at each extremity. We hypothesize that these charged residues are involved in anchoring to the membrane lipid headgroups. The proline residue that defines the break between the two helices of S3a and S3b of Kv1.2/2.1 is absent in A3 channels, suggesting that S3 might have a continuous helix.

### Electrophysiological Characterization of the cA3 Subunit

**Figure 2:**
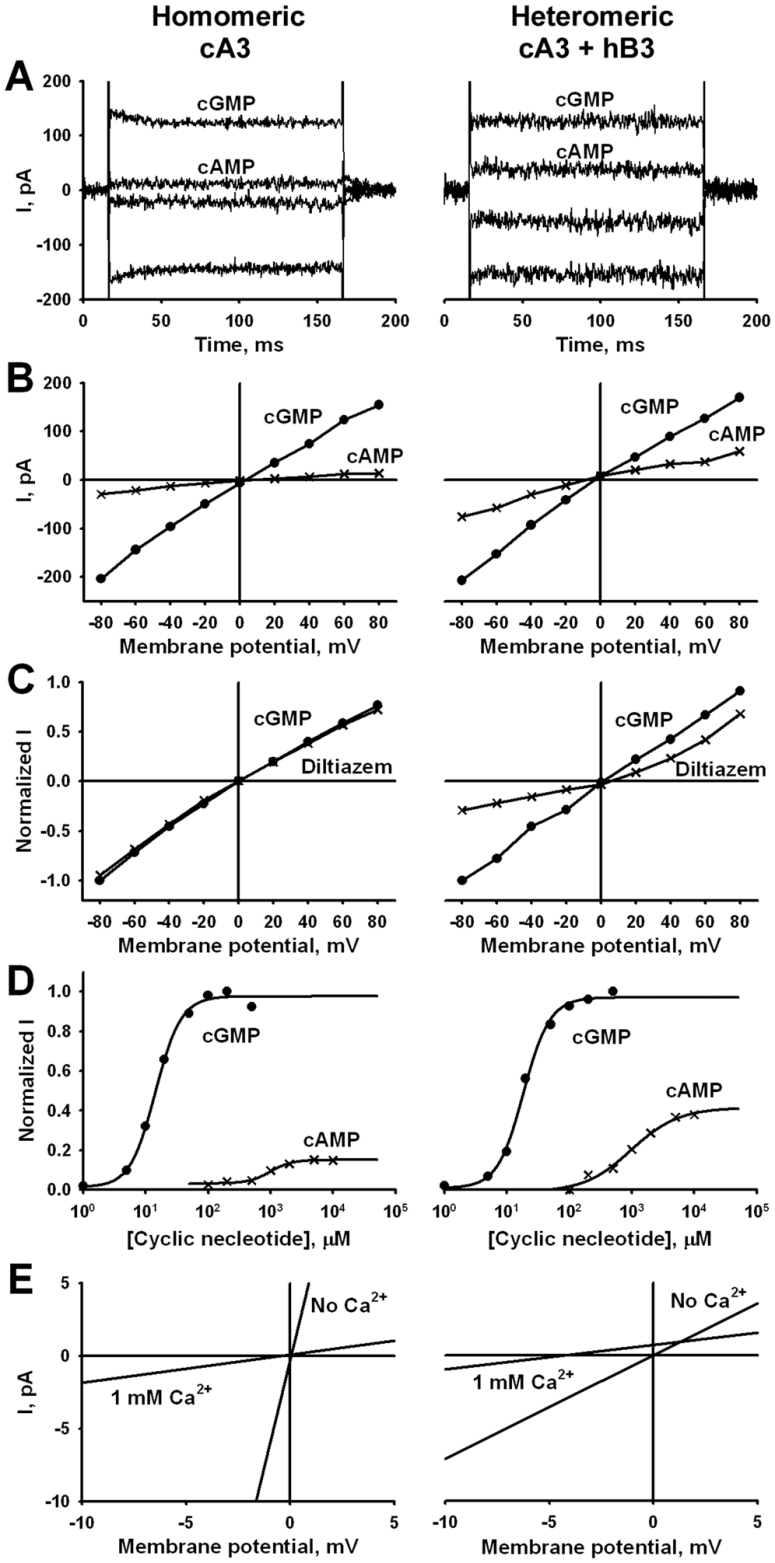
Characterization of CNG channels formed from cA3 alone or co-expressed with hB3. Recordings from cA3 channels (homomeric) are shown in the left panels and co-expression with hB3 subunits (heteromeric) are shown on the right. Net currents are shown after subtraction of currents measured in the absence of nucleotides. **A. Nucleotide-activated currents show cAMP efficacy.** Currents were activated with 200 µM cGMP and 5000 µM cAMP at −60 mV (negative currents) and 60 mV (positive currents). The cAMP efficacy is 0.14 for homomeric channels and 0.38 for hetromeric channels. **B. IVs with 200 µM cGMP or 5 mM cAMP at ±80 mV.** Both the electrode and bath solutions have EDTA and EGTA to eliminate the characteristic Ca^2+^ block of Na^+^ current in CNG channels. In the absence of divalent cations, a slight inward rectification is observed with a 1.6 fold larger current at −60 mV compared to 60 mV for cGMP and 2.2 fold for cAMP in homomeric channels. Little change is seen in heteromeric channels containing the human B3 subunit with rectification of 1.25 fold for cGMP and 1.7 fold for cAMP. **C. L-** ***cis*** **diltiazem block is greater in presence of hB3 subunits.** IVs were recorded with 100 µM cGMP alone or with 25 µM L-*cis*-diltiazem added to the bath. Net currents were normalized to the maximum cGMP activated current for each patch. **D. cGMP and cAMP dose response relationships.** Each plot shows the ligand concentration-dependent activation of homomeric or heteromeric channels at −60 mV. Data were taken from a single patch. The K_0.5_ for cGMP is 14.2 µM for homomeric and 18.6 µM for heteromeric channels; the N_h_ is 2.1 for both channel types. The cAMP K_0.5_ values are 947 µM and 966 µM, respectively with N_h_ values of ∼2.5. **E. Current reversal potential shifts (E_rev_) with 1 mM [Ca^2+^]_i_ at 200 µM cGMP.** Each panel shows net currents activated by 200 µM cGMP with and without 1 mM [Ca^2+^]_i_. Voltage ramps were −20 mV to 20 mV. The E_rev_ shift of homomeric channels is −0.47, whereas that of the heteromeric is −4.02.

We expressed homomeric cA3 or heteromeric cA3+ hB3 channels in tSA201 cells to characterize the electrophysiological properties. Substituting the hB3 for the cB3 is reasonable since an appropriate expression vector containing the *cB3* was not available. Further, the *hB3* gene was used successfully for retinal gene augmentation therapy to restore cone function in dogs with *CNGB3*-achromatopsia confirming proper functioning of cA3+ hB3 channels [41]–[43]. Macroscopic currents were recorded from inside-out patches in the range of 200–800 pA with exposure to bath solutions containing cGMP or cAMP. Figure 2A shows a representative patch with nucleotide activation at saturating concentrations of 200 µM cGMP or 5 mM cAMP. Cyclic AMP is a weak agonist in cone channels; the fraction of maximal current established with saturating concentrations of cGMP increases significantly with co-expression of the hB3 subunit. Averaged data are summarized in Table 1 and compared with previous data from human cone CNG channels.

**Table 1:** Comparison of canine CNG channel properties to human CNG channels.

cGMP		cAMP		I_cAMP_/I_cGMP_	I_dil_/I	References
K_0.5_ (µM)	N_h_	K_0.5_ (µM)	N_h_			
**cA3**						
10.3±1.24 (7)	2.40±0.33	1665±226 (7)	2.06±0.30	0.19±0.01 (16)	0.79±0.05 (4)	This work
**hA3**						
13.5±2.5 (39)*	2.2±0.2	1329±3.69 (39)	1.3±0.2	0.12±0.04 (39)	0.94±0.04 (6)	[6]
12.9±0.5 (62)*	2.1	1212±37 (53)	1.4	0.12±0.01 (48)		[25]
8.20±0.34 (7)	1.90±0.13	580±110 (10)	1.39±0.37			[69]
11.1±1.0 (13)	1.83±0.19					[24]
8.63±0.81 (21)	1.53±0.17					[26]
**cA3+ hB3**						
16.0±1.05 (6)	1.87±0.15	1569±262 (6)	2.36±0.66	0.33±0.02 (25)	0.40±0.02 (8)	This work
**hA3+ hB3**						
19.9±3.8 (56)*	2.0±0.2	897±1.77 (56)	1.6±0.2	0.27±0.09 (59)	0.26±0.11(15)	[6]
15.8±0.3 (61)*	2.0±0.2	846±33 (54)	1.5±0.3	0.36±0.01 (48)		[25]
19.4±1.6 (6)	1.65±0.18	570±60 (6)	1.03±0.13			[69]
26.2±1.9 (16)	1.82±0.11					[24]
18.2±1.84 (13)	1.22±0.15					[26]

The K_0.5_ values, Hill coefficients (N_h_), cAMP efficacy (I_cAMP_/I_cGMP_), and L-cis Diltiazem block (I_dil_/I) of homomeric and heteromeric channels were determined at −60 mV. Averaged calcium to sodium permeability rations were calculated to be 1.52±0.52 (n = 5) for cA3 and 17.7±6.4 for A3+ B3 (n = 4). Comparison permeability ratios for hA3 were not available. Statistical values were SEM except for the two reports indicated with an * which were SD values.

In order to maximize currents recorded from CNG channels and facilitate characterization of the CNG channel properties, divalent cations are chelated in both the bath and electrode solutions. The hallmark calcium block of cone channels, therefore, is eliminated in our recordings [44], [45]. In the absence of divalent block, the current-voltage (IV) curves are nearly linear. Representative IVs at saturating concentrations of cGMP and cAMP are shown in Figure 2B. The lack of voltage-dependent activation in CNG channels is in contrast to channels in the larger family of *Shaker* channels which are activated by membrane potential changes.

Figure 2C characterizes pharmacological block by L-*cis*-diltiazem in channels activated by 100 µM cGMP. The current block is pronounced in channels expressing the hB3 subunit compared with homomeric cA3 channels; the block increases from <10% block with homomeric channels at −60 mV, to ∼70% block in heteromeric channels expressing hB3 subunits. Together, the cAMP efficacy and L-*cis*-diltiazem block provide a rapid evaluation of each patch as to whether the currents reflect homomeric,(low cAMP efficacy and very little L-*cis*-diltiazem block), or heteromeric channels (higher efficacy and significantly greater block) [6], [25]. Therefore, we routinely measured the cAMP/cGMP efficacy and the L-cis-diltiazem block in patches from cells transfected with both *cA3*+ *hB3*. Approximately 20% of patches transfected with *cA3*+*B3* exhibited homomeric channel characteristics; these patches were discarded for further analysis of heteromeric channel properties (see Discussion for possible relevance of this observation).

Figure 2D shows cGMP and cAMP dose-response relationships. The cooperative nucleotide activation is apparent although cAMP dose-response curve fitting is difficult because of the high concentrations needed to saturate the currents and the small relative currents. The K_0.5_ for cGMP increases slightly from 14.4 to 18.4 µM with co-expression of the hB3 subunit; the K_0.5_ for cAMP is ∼1 mM for both channel types. The Hill coefficients of the fits to these data are in the range of 2.0–2.5, similar to values measured for hA3 channels (see Table 1).

We examined the relative calcium to sodium permeability using reversal potential (E_rev_) shifts following a single-sided addition of 1 mM CaCl_2_ to the bath (intracellular side). Figure 2E shows a representative IV in the presence of 200 µM cGMP with and without 1 mM [Ca^2+^]_i_. The reversal potential shifts to more negative values in both homomeric and heteromeric channels in the presence of [Ca^2+^]_i_. The E_rev_ of the heteromeric channel has a shift of −4.02 mV compared to −0.47 mV for homomeric channels. The mean P_Ca_/P_Na_ ratio (see Methods) of the homomeric cA3 channels is 1.5, whereas that of heteromeric channel is 17.2. Possible implications of the difference in Ca^2+^ permeability between homomeric and heteromeric channels for affected daylight-blind *B3*-mutant dogs are considered in the Discussion.

### Loss of Channel Function and Mislocalization with Asp/Asn Mutation at D3 of the *Tri-Asp Motif*

**Figure 3:**
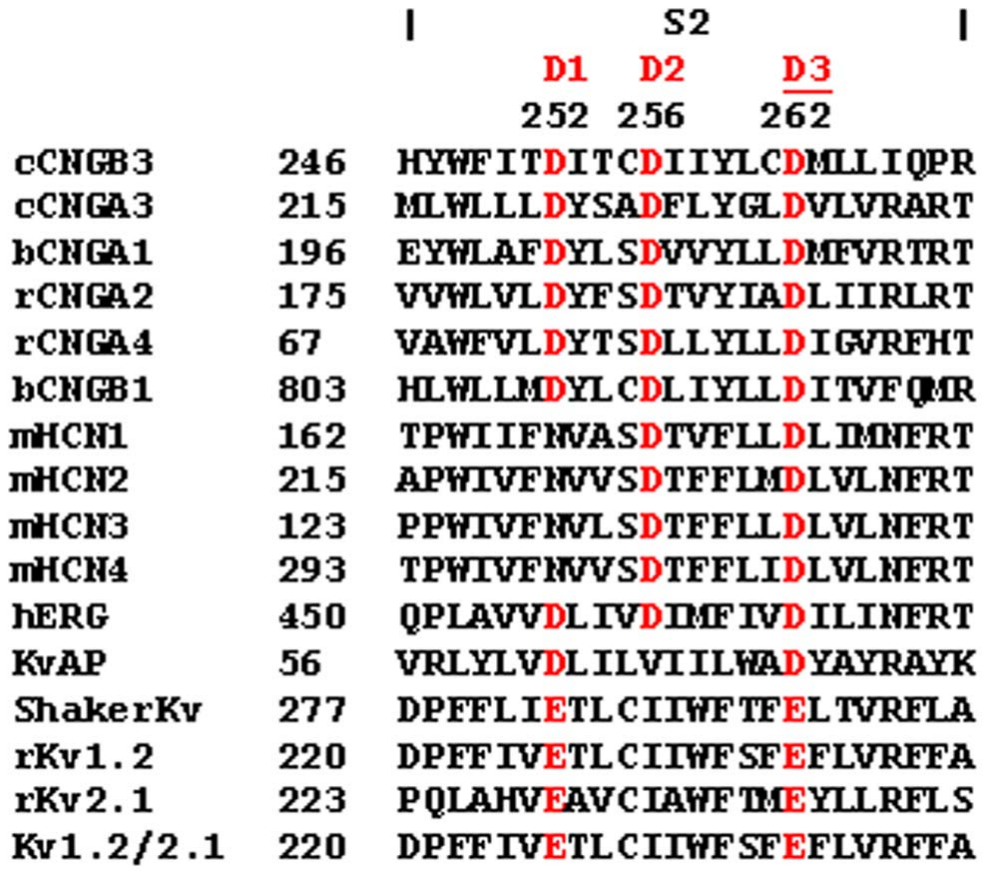
Sequence alignment of the S2 domain of the cB3 subunit residues define the *Tri-Asp motif*. Asp252, 256 and 262 in canine B3 delineate the highly conserved *Tri-Asp motif* which is conserved in all CNG channels with relative positions 2 and 3 conserved in the HCN channels. The flanking positions 1 and 3 are acidic residues in the Kv channels aligned here. c = canine; b = bovine; h = human, r = rat; m = mouse. CNGA1 and CNGB1 are rod channels; CNGA2 and CNGA4 are channel subunits expressed in olfactory cilia.

An alignment of an S2 helical region of CNG channels with related Shaker superfamily channels is shown in Figure 3. The Asp262 (denoted as D3) residue of the cB3 subunit is located at the C-terminal end of the S2 helical region. Two other Asp residues, D252 (denoted D1) and D256 (denoted D2), are N-terminal to D3; this short region with three acidic residues is conserved amongst CNG channels and is designated the *Tri-Asp motif*. Within the larger family, the hyperpolarization-activated cyclic nucleotide-gated (HCN) channel isoforms conserve Asp residues at D2 and D3 positions and the Shaker K^+^ family members shown here retain acidic residues at the two flanking positions.

**Figure 4:**
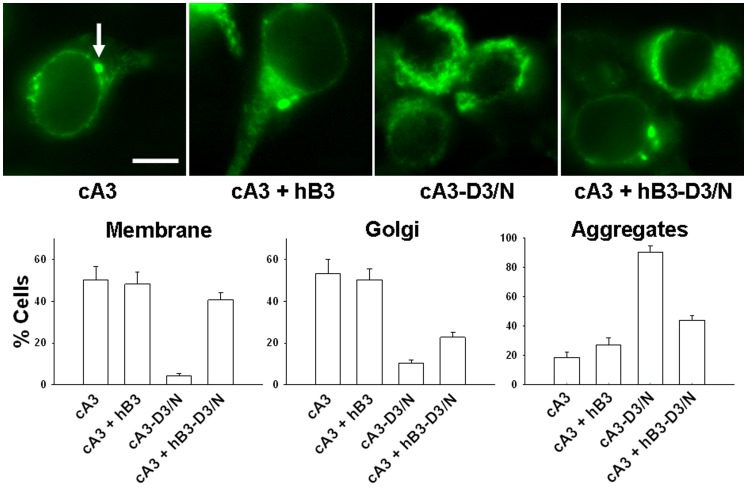
Cellular localization of YFP-tagged cA3 constructs. Upper panels: Micrographs illustrating expression of cA3, cA3-D3/N, cA3+ hB3, and cA3+ hB3-D3/N; the arrow indicates a Golgi-like organelle. Intracellular aggregates are seen in all but the cA3-Y cells. Lower panels: averaged expression characteristics constructed from counts of >800 cells. The cA3 and cA3+hB3 expression patterns have a small but significant increase in intracellular aggregates (p = 0.02 on unpaired t-test). Clear, significant differences are seen with expression of mutant D3/N subunits although see text for the interpretation of the cA3+ hB3-D3/N mutant subunit. Scale bar 10 µm.

Based on the importance of the *Tri-Asp motif* in cB3 and the conservation of D3 (Asp231) in cA3, we generated a mutant cA3 subunit cA3-D3/N. Loss of nucleotide-activated currents was apparent in patches from cA3-D3/N homomeric channels as indicated in Table 2. This loss of function of cA3-D3/N channels might be due to improper protein synthesis and folding, defective membrane targeting or improper subunit assembly. Protein mislocalization arising from folding defects is a well-recognized cause of channelopathies [46]. The presence of a fluorescent tag on the cA3 subunit provided a way to investigate cellular localization of the CNG channels in heterologous expression studies. Figure 4 shows the expression of YFP-tagged cA3 subunits in individual cells; averaged data are shown in the histograms. The large Golgi-like organelles and the membrane fluorescence are typical of functional channels in heterologous expression systems. Further, few intracellular aggregates are apparent in cells expressing wild-type subunits. The localization of the cA3-D3/N homomeric channels show very few cells with membrane or Golgi fluorescence and nearly 100% of cells express intracellular aggregates consistent with a protein folding defect.

**Table 2:** Nucleotide-activation of channels comparing wild-type and mutant subunits.

	cA3	cA3+ hB3	cA3–D3/N	cA3+ hB3–D3/N
**cGMP Responses**	100% (33)	100% (25)	0% (15)	60% (10)
**cAMP Responses**	100% (33)	100% (25)	0% (14)	60% (10)
**I_max_cA/** **I_max_cG**	0.19±0.01 (33)	0.33±0.02 (25)	N/A	0.15±0.01 (4)

The cAMP efficacy of patches from co-transfected cA3+ hB3-D3/N cells is consistent with these cells expressing homomeric cA3 channels supporting the conclusion that these channels express only the cA3 subunits.

When hB3 is co-expressed with cA3, the cellular localization data shows a small but significant increase in the fraction of cells expressing intracellular aggregates (Figure 4). Unexpectedly, the aggregate fluorescence data with cA3+hB3-D3/N show a reduction in the fraction of cells expressing intracellular aggregates compared to cA3-D3/N. The micrograph in Figure 4 illustrates one of the two cells shown in the far-right panel expressing Golgi with no intracellular aggregates; the other cell expresses only aggregates. This fluorescence pattern is consistent with the electrophysiology data in which ∼60% of the patches matched the characteristics of homomeric channels and ∼40% of the patches presumably contained nonfunctional, heteromeric channels. Collectively, we surmise that homomeric cA3 channel assembly competes with heteromeric assembly when the hB3-D3/N mutant is expressed.

### Loss of Channel Function and Mislocalization with other *Tri-Asp motif* Substitutions

The strict conservation of the *Tri-Asp motif* in CNG channel subunits prompted us to generate other mutations in the *Tri-Asp motif* of A3 where we could investigate function in a homomeric channel. In previous experiments, Asp substitutions D1/C, D1/E, D2/I, D2/C, D2/N, D2/E and D3/E, were examined in hA3 [47]. All of these mutations resulted in the loss of channel activity and mislocalization. The summary in Table 2 is consistent with the hypothesis that all Asp residues of the *Tri-Asp motif* are required for proper channel biogenesis and function [48].

### Structural Insights about the S1–S6 Region of CNGA3 Channels from Homology Modeling and Molecular Dynamics (MD) Simulations

To date, no high-resolution structure of the TM region of a eukaryotic CNG channel is available. As an alternative, to gain insight into the role of the *Tri-Asp motif* and, in particular, to gain insights about possible interactions involving D3 side chains, we built a model of the TM portion of cA3. We used the Kv1.2/2.1 high resolution structure [40] as a template for homology modeling and we relaxed the model in its lipid environment using molecular dynamics (MD) simulations.

A major difference between cA3 and Kv1.2/2.1 is the large number of charged residues located in S2–S4 (12 positive and 9 negative charges) of cA3, compared to Kv1.2/2.1 (8 positive and 5 negative charges). In Shaker channels, crystal structures have shown that these charged residues are shielded away from the unfavorable lipid environment and are involved in salt bridge pairings pointing towards the center of the solvated 4-helical bundle. Accordingly, to build the homology model, we enforced distance restraints between pairs of positively and negatively charged residues (details in Materials and Methods). Because the proline residue that defines the break in the S3 helix of Kv1.2/2.1 is absent in cA3 channels, we enforced helicity of the S3 segment thereby directing the charges of S2–S4 towards the hydrated lumen of the four helix bundle. These constraints were initially imposed during the equilibration phase of the MD simulations and gradually released.

**Figure 5:**
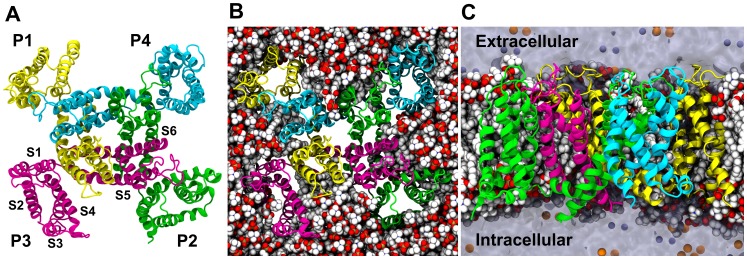
Structural model of the cA3 homotetramer in a lipid environment. A. Top view: The four subunits are represented as ribbons. P1 is colored in yellow, P2 in green, P3 in pink and P4 in cyan. Side chains are omitted for clarity. The tetrameric assembly of the two N-terminal helices S5 and S6 forms the central pore. The other TM segments (S1–S4) of each subunit forms a bundle located at the periphery of the pore. **B. Top view:** The protein is represented as in A. The POPC lipids embedding the channel are represented as Van der Waals spheres, with carbons in white, phosphates in brown, oxygens in red and nitrogens in blue. Water molecules and ions are not shown for clarity. **C. Side view with protein and lipids added.** The contours of the water-filled volume are represented as a transparent surface. The Na^+^ ions are in orange and the Cl^-^ in purple. The extracellular domain is located at the top and in the intracellular domain is located at the bottom.

The model shows the tetrameric assembly of S5–S6 defining the pore and S1–S4 defining four auxiliary domains (Figure 5A) distributed around the pore assembly. The central axis of the pore defines the relatively non-selective conduction pathway for cations. Figures 5B and C show the arrangement of the lipid bilayer membrane around the tetrameric channel and highlight the role played by the membrane in stabilizing the tertiary and quaternary structure.

**Figure 6:**
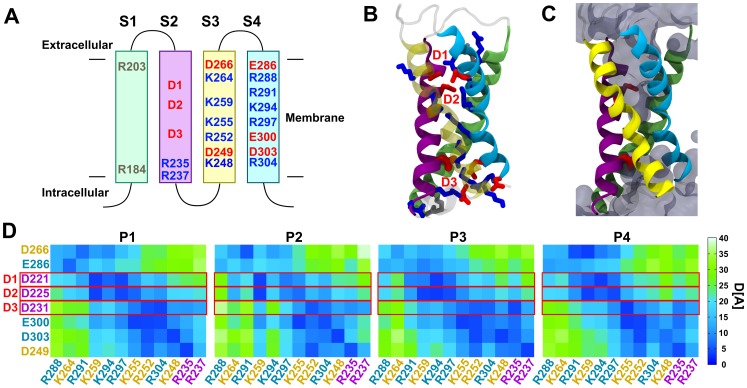
*Tri-Asp motif* in S2 and charged residues of the S1–S4 bundle. A. Topology of the S1–S4 domain of cA3 subunit and localization of the charged residues. S1 is represented in green, S2 in purple, S3 in yellow and S4 in cyan. Charges not involved in salt bridging are shown in grey, other positive K and R are shown in blue, negative D and E are shown in red. D1, D2 and D3 correspond to D221, D225, and D231 in cA3, respectively. **B. Molecular model of a representative S1–S4 domain with all charges of the bundle represented.** Color coding is the same as in B. Positive charged side chains charges are in blue, negative charged side chains in red. The *Tri-Asp motif* residues are noted as D1, D2 and D3 in red. Note that this is a snapshot of the salt bridge network of the P4 subunit voltage sensing domain. Lipids, water, and ions are omitted for clarity. **C. Same as B with the addition of water.** The Tri-Asp motif residues are depicted as red rods, the solvent accessible volume is delineated by a transparent surface. **D. Mapping of the average distance between negative and positive residue pairs.** The atom, NZ, is used for K, CZ for R, CG for D, and CD for E. Each subunit is presented separately. The distances were averaged over the last 20 ns of simulation. Distances range from 0 (deep blue) to 40 Å (light green). The charge labels are colored according to the TM segment they belong to. Note that the salt bridge network is rather different from one subunit to the other, highlighting the presence of several conformational changes of similar stability.

As Figure 6A shows, the *Tri-Asp motif* is located on the S2 segment of the S1–S4 bundle. D3 is the intracellular-most charge. The rest of S2, S3, and S4 also contain a large number of charges of both signs. Figure 6B reveals that all the charges are stable pointing towards the center of the domain. As in Shaker channels, they are stabilized in the TM position through salt bridge pairings. Such a pairing mechanism is thought not only to be important for function but also for proper folding.

It is apparent that the charges are organized in two separate clusters: the extracellular cluster contains D266, E286, D1 and D2 as negative charges and R288, K264, R291, K259, K294 and R297 as positive charges; the intracellular cluster contains D3, E300, E303 and D249, and K255, R252, R304, K248, R235 and R237. Interestingly, the topology of the solvent accessible volume is shaped as an hourglass and the simulation reveals water molecules protruding from the extracellular and from the intracellular medium (Figure 6C). The two clusters of charges are then solvated by the extra- and the intra-cellular half, respectively. Compared to Shaker channels in which the charge composition only allows a single specific salt bridge network, here, due to the larger number of positive and negative charges and to their spatial proximity, several different salt bridge pairings can be envisioned. Figure 6D reports the time-averaged distances between all possible pairs of positive and negative charges during the last 20 ns of the simulation and highlights the diversity of salt bridge pairings in the S1–S4 bundles. It demonstrates that, despite enforcing four-fold symmetry of these salt bridges during homology modeling and the initial steps of the equilibration, the S1–S4 bundles of the four different subunits rearranged along the simulation until each reached a different salt bridge pairing pattern. The simulations thus show that 1) several conformations, each characterized by a different salt bridge network, are stable on the tens of nanosecond timescale and 2) transitions between different conformations are possible at room temperature. Note also that we cannot rule out the possibility that other conformations exist and could be explored in other simulations. Altogether, contrary to Shaker channels, it is not possible to characterize the structure of the S1–S4 bundle by a single well-defined network of salt bridges.

Note that while the *Tri-Asp motif* is conserved in all CNG subunits (Figure 3), and specifically in cB3, the charges in the other TM segments are not. Specifically, the positive charge R235 in cA3 is lost in cB3 (I266); R252 in cA3 is reversed to a negative residue (E284) in cB3; R297 in cA3 is S328 in cB3 (Figure 1), among others. While we do not expect the details of the cA3 structural model to be conserved in cB3, because of the sequence similarity, we think that the coarse features of the vestigial voltage-sensing domain can be extrapolated to understand the effect of the D3N mutation in canine B3.

## Discussion

In this work, we investigated a highly-conserved region in the S2 TM segment of the cB3 channel subunit, the *Tri-Asp motif* which contains Asp262, the residue mutated to Asn in German shorthaired pointer dogs affected with achromatopsia [22]. To examine the effect of the missense B3-D3/N mutation in a homomeric channel, we expressed the D3/N mutation in the equivalent position in cA3. The mutation results in a loss of channel function and mislocalization of a fluorescent-tagged subunit consistent with a misfolded subunit. The evolutionary conservation of the *Tri-Asp motif* in all CNG channel subunits is consistent with a critical role for these acidic residues in function, folding and subunit assembly.

### Proposed “Zipper” Model for S2 Membrane Threading During CNG Channel Synthesis

Our experimental results can be considered in light of previous work with voltage-gated K^+^ channels. In Shaker and hERG channels, alterations in an acidic residue at either position 1 or 3 in the motif alter but do not eliminate channel function [49]–[51]. The loss of function with a mutation at position 1, 2 or 3 in cA3 channels reveals clear differences in the role of these acidic residues between CNG channels and the related Shaker K^+^ channels.

Insights about the role of the S2 acidic residues in folding were revealed in studies with the K^+^ channel family. In Shaker family, the interactions between charged residues of S2–S4 are important to stabilize these helices [52]–[55]. A similar mechanism was reported in sodium selective channels of the same six TM-segment architecture [56]. Recently, interactions between the acidic residues in D1 and D3 position and positive residues of S4, have been shown to mediate folding [57] and membrane insertion in both the plant Kv-like channel KAT1 and in Shaker [58]–[60]. In the folding model for Shaker channels inferred from the recent studies, folding of the voltage-sensing domain involves a two-step mechanism. First, the uncharged helix S1 is inserted into the membrane. Next, charged amino-acids from S2–S3–S4 are paired to form a three helix bundle. Finally, this assembly is released from the translocon into the membrane.

Our folded cA3 channel model predicts that the side chain interactions of the highly-conserved D3 are required for inter-helical contact through salt bridge pairing. Also, D1 and D2 contact the positive residues of S2–S4, similar to what is observed in Shaker channels. We predict therefore that a mutation of D1, D2 or D3 to an uncharged residue leads to the loss of salt bridges. Although modeling the unfolding of a membrane protein is computationally inaccessible on the timescale achievable by MD simulations, destabilization of the folded state of the S1–S4 bundle is our current understanding of changes introduced by mutations at D3.

Moreover, our experiments involving D/E mutations show that not only charge conservation is important, but also side chain length. Our finding is consistent with the previous work cited above on Shaker folding that conserving the negative charge on the D1 and D3 residues affects folding [60]. Unlike for channel function where charge conservation might alter but not eliminate function, during protein folding, the conservation of the negative charge might not be sufficient to stabilize the nascent S1–S4 bundle; the side chain length might be critical as well. Considering the sequence and structural similarity between Shaker family members, we argue that any mutation of D3 would likely lead to protein misfolding.

### Why Does a Missense Mutation or the Deletion of CNGB3 Result in Dayblindness?

If A3 subunits can form homomeric functional channels, why do *B3*-mutant dogs not substitute A3 channels? In other words, how does the B3 subunit affect A3 expression, subunit assembly and channel localization? Further, to what extent are heterologous expression systems appropriate models for CNG subunit assembly and outer segment localization in cones? We know that a C-terminal leucine zipper (CLZ) in CNG channel A-type subunits directs subunit assembly. An elegant investigation of the CLZ domain by Zagotta and colleagues, using intact channels as well as soluble constructs from both CNGA1 (A1) and A3, provides insight and raises deeper questions [3]. The soluble CLZ domains were crystallized and the structures revealed long, parallel, three-helix, coiled-coil domains. The trimeric association of A1 subunits attracts a CNGB1 (B1) subunit N-terminal domain [12]–[14] resulting in a 3∶1 A1:B1 ratio for rod channels. The A3 CLZ domains may not follow a high affinity association with B3 because published data show both a 2∶2 [11] and 3∶1 A3:B3 subunit composition in cone CNG channels [3], [10]. Thus, the A1 and A3 CLZ domains might encrypt somewhat different information with regard to B-type subunit recruitment. Also related to subunit recruitment, olfactory CNG channels have a 2∶1∶1 subunit composition with A2:A4:B1b and both A2 and A4 have CLZ domains [61]. In summary, questions remain about how information in the CLZ sequences of these related subunits direct subunit assembly. Future biochemical and heterologous expression studies likely will reveal additional insights about CNG subunit selectivity and interaction interfaces, however, understanding how the cone regulates channel assembly and directs channel insertion might require a deeper understanding of cell-specific regulatory check points in subunit assembly.

A related insight about CNG subunit assembly comes from our co-transfection experiments with cA3 and hB3. Approximately 20% of patches excised from cells co-transfected with cA3+ hB3 express homomeric-type currents. By contrast, Yau and collaborators co-transfected hA3 and hB1 in ratios from 25% hB1 to 5 times more hB1 and reported nearly 100% of the currents were heteromeric [10]. Consistent with that finding, Varnum and collaborators investigated the hA3 and hB3 subunit assembly in oocytes varying the mRNA ratios 10 fold [11]. Their patches showed heteromeric channel properties independent of the mRNA ratios. Our work then could reflect a species difference since we monitored cA3 co-assembly with hB3 and the previous studies expressed subunits from the same species. However, additional insights about subunit association were revealed with co-expression of cA3 with hB3-D3/N subunits. While we expected a loss of channel activity in cells co-expressing a mutant B3 subunit, ∼60% of patches showed homomeric-type currents a result supported by the cellular localization studies showing a mix of the normal phenotype (membrane and Golgi-like features) and the mutant phenotype (intracellular aggregates). Thur, we infer that misfolded B3-D3/N subunits alter the cA3 subunit association bias in favor of the assembly of functional A3 homomeric channels in heterologous expression systems. Again, the question arises as to whether heterologous expression reflects channel assembly in the cone cells.

In order to probe CNG subunit expression and assembly in normal and affected cones, one would like to investigate individual cone function directly using electrical, immunological and biochemical measurements. Previous work on catfish cones defined the CNG channel activation and electrophysiological properties [42], [44], [62] which were later matched to those of heterologously expressed cone channels when the genes were available for study. More recently, Korenbrot and collaborators contributed significantly to our understanding of cone physiology using striped bass cones [63]–[65] but unfortunately, these studies have limited application to understanding human channelopathies.

The spontaneously-occurring inherited canine diseases closely resemble achromatopsia in humans and provide valuable models for developing human gene therapies [41], [42], [66]. A spontaneously-occurring genomic deletion of the *B3* gene was identified in a canine breed resulting in dayblindness [22]. In the affected *B3*-deletion dogs and based on the increased Ca^2+^ permeability conferred by the B3 subunit (this work), we suggest that homomeric A3 channels in the outer segments would alter Ca^2+^ influx significantly. Although we have no direct information on Ca^2+^ homeostasis in canine retinas, previous studies examining Ca^2+^ homeostasis in photoreceptors showed an increase in [Ca^2+^]_i_ in cone dystrophies and apoptotic cell death in photoreceptors [67], [68] highlighting the potential for aberrant [Ca^2+^]_i_ levels to promote pathophysiology.

Experiments using retina homogenates from *B3*-deletion mutant dogs showed normal expression levels of cA3 protein however, no A3 immunohistological staining was observed in the cone outer segments raising the possibility that although A3 subunits are synthesized, they are not trafficked to the outer segment membranes [42]. This observation suggests that a wild-type B3 subunit is required for proper targeting of A3 subunits to the outer segment. In our previous work, the D262N missense mutant dog retinas show that cA3 outer segment expression is recovered following gene therapy with the *hB3* gene [42]. Further, since cA3 subunits have an increased propensity to form homomeric channels in the presence of a misfolded mutant B3 subunit (our heterologous expression data), are functional A3 channels expressed in missense mutant dogs? Future studies of CNG channel subunit expression, composition and membrane targeting *in vivo* might integrate these questions using immunohistochemistry and other approaches in normal and affected retinas.

In summary, channelopathies associated with missense mutations draw attention to amino acids important in our quest to understand channel structure-function properties. The insights gained from biophysical and computational studies of mutant channels contribute to our understanding of the channel structure/function relationship and raise intriguing questions about the role of particular amino acids in subunit formation and channel biogenesis. Nevertheless, despite the challenges of relating our understanding of channelopathies across disciplinary approaches from molecular models to gene therapy, the application of these approaches provides future promise for a deeper molecular understanding of the diseases.
